# Continuous blood purification in patients with pheochromocytoma crisis: A case report

**DOI:** 10.1002/ccr3.8036

**Published:** 2023-10-19

**Authors:** Meinong Zhong, Yuanqiang Zhu, Shaofang Wang, Hengying Fang, Guili Chen

**Affiliations:** ^1^ Division of Urology The Third Affiliated Hospital of Sun Yat‐Sen University Guangzhou China; ^2^ Internal Medicine Intensive Care Unit The Third Affiliated Hospital of Sun Yat‐Sen University Guangzhou China; ^3^ Department of Nursing The Third Affiliated Hospital of Sun Yat‐sen University Guangzhou China

**Keywords:** cardiogenic shock, case report, continuous blood purification therapy, efficacy, multiple organ dysfunction, pheochromocytoma crisis

## Abstract

**Key Clinical Message:**

Pheochromocytoma crisis accompanied by multi‐organ failure necessitates prompt and comprehensive interventions, including VA‐ECMO, CRRT, and others. Successful laparoscopic tumor resection promotes favorable outcomes and recovery.

**Abstract:**

Pheochromocytoma crisis is commonly associated with high mortality, high surgical risk, and rapidly fatal complications. This article presented successful treatments and nursing experiences in a patient with pheochromocytoma who developed cardiogenic shock and multiple organ failure. We report a case study of a 32‐year‐old female patient who experienced pheochromocytoma crisis accompanied by multiple organ failure. Initial assessment of bedside echocardiography revealed an extremely low left ventricular ejection fraction of 8%. The patient was promptly resuscitated though tracheal intubation and venoarterial extracorporeal membrane oxygenation (VA‐ECMO), in conjunction with continuous renal replacement therapy (CRRT), alpha‐blockers, beta‐blockers, and other pharmacological interventions to manage blood pressure and heart rate. These interventions resulted in a remarkable increase in the left ventricular ejection fraction of 67%. However, the patient subsequently developed severe sepsis, which may have been caused by the intubation procedure, necessitating the discontinuation of VA‐ECMO while maintaining CRRT. Close monitoring of plasma catecholamine metabolite level, hemodynamic index, inflammatory marker, liver and kidney functions, and electrolytes during CRRT support allows for evaluating the efficacy of these measures and assessing the impact of CRRT on pheochromocytoma crisis. Eventually, the patient successfully underwent laparoscopic resection of a large pheochromocytoma, leading to favorable prognosis and a successful recovery. Continuous blood purification therapy can effectively eliminate catecholamines and their byproducts from the plasma, stabilize hemodynamics, improve heart, liver, and kidney functions, significantly reduce inflammatory cytokine levels significantly, and extend the surgical window for patients.

## Graphical abstract

Results of renal CT examination. The arrow points to the left adrenal mass, the size is 5.345.34 × 5.73 cm.

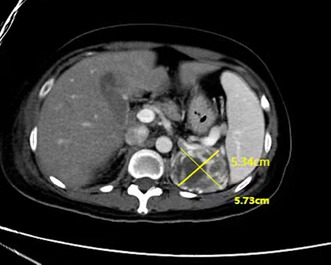

## 1 INTRODUCTION

Pheochromocytoma arises from the chromaffin cells of the adrenal medulla and can result in excessive secretion of catecholamines. This can trigger a pheochromocytoma crisis, which is associated with a high mortality rate up to 13%.^1^ Catecholaminergic cardiomyopathy induced by pheochromocytoma crisis may lead to damage or dysfunction in multiple organ system, myocardial infarction, acute left ventricular failure, cardiogenic shock, and other fatal outcomes, particularly in cases where the pheochromocytoma is undiagnosed or poorly controlled.^2^ Surgical resection is the primary treatment for pheochromocytoma, however, the instability of perioperative hemodynamics poses a high risk for anesthesia and presents significant treatment challenges.^3^ Currently, research on the diagnosis, treatment, and nursing care of such patients is mainly based on case reports. Venoarterial extracorporeal membrane oxygenation (VA‐ECMO) has been reported to stabilize circulation and extend the surgical window for patients with severe myocardial injury or circulatory failure.^4^ In June 2021, we successfully treated a patient with pheochromocytoma who presented with cardiogenic shock as the initial manifestation of multiple organ failure. This case report details our experience in the diagnosis, treatment, and nursing care of pheochromocytoma with various severe complications.

## 2 CASE PRESENTATION

### 2.1 Case data

**FIGURE 1:**
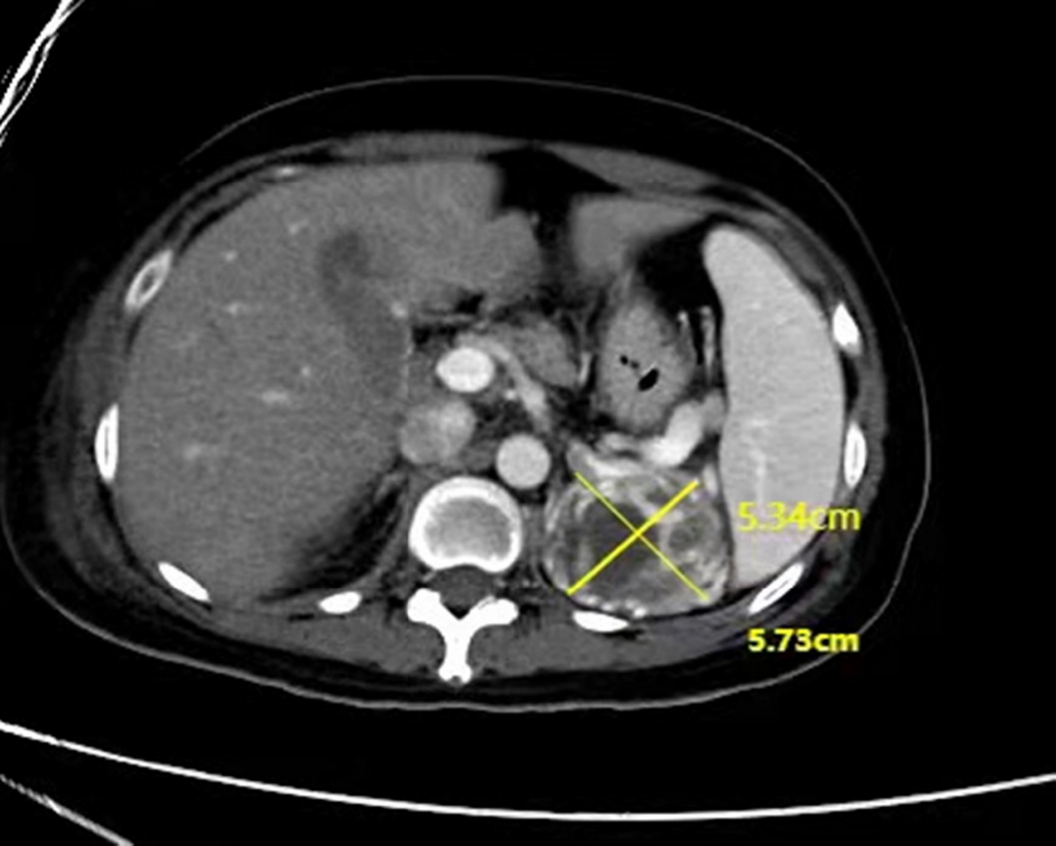
Results of renal CT examination. The arrow points to the left adrenal mass, the size is 5.34 × 5.73 cm.

**FIGURE 2:**
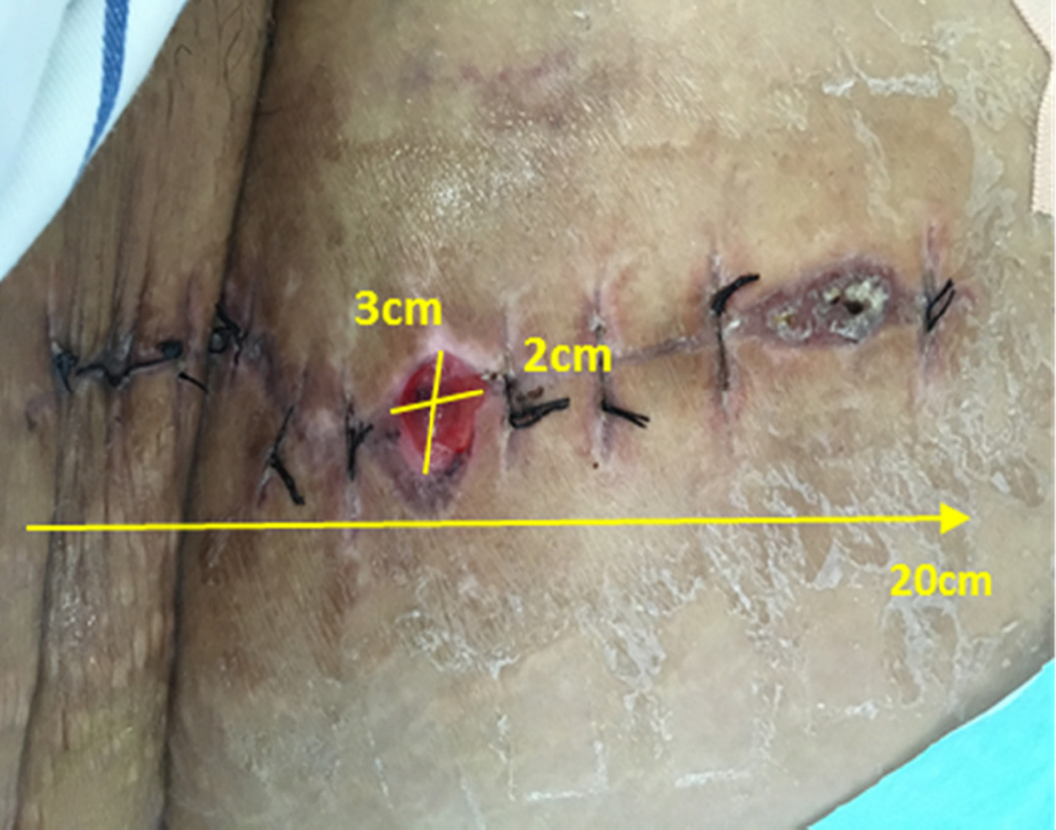
Picture of the patient's right thigh wound. The arrow points to the right thigh surgical wound, about 20 cm long, the size of the rupture is about 2 × 3 cm.

A previously healthy 32‐year‐old woman experienced a sudden choking cough while eating ice cream on May 8, 2021. This caused progressive chest tightness, shortness of breath, and dyspnea. The patient was diagnosed with severe pneumonia, acute left heart failure, cardiogenic shock, and a giant pheochromocytoma (Figure 1). Initial treatment at a local hospital included tracheal intubation, VA‐ECMO combined with CRRT treatment, α‐blockers (phentolamine), β‐blockers (propranolol), and other supportive care. After 10 days of treatment, the left ventricular EF increased from 8% to 67%, and ECMO support was withdrawn. Right femoral vein vascular repair was performed (Figure 2), and CRRT was maintained, but the patient developed sepsis. The patient was then transferred to our hospital on June 3 for further treatment. During the subsequent treatment at our hospital, anti‐infection, anti‐heart failure, volume expansion, CRRT, phentolamine, propranolol, lyophilized recombinant human brain natriuretic peptide, and morphine were administered. Meticulous wound care was also provided. CRRT treatment was suspended on June 9. On June 15, the patient was transferred to the general ward for further preparation but experienced another pheochromocytoma crisis during the bumpy transfer, resulting in multiple organ failure and malignant arrhythmia. Prompt intervention, including nasal high‐flux oxygen therapy, CRRT, and other treatments, stabilized the patient's condition. On June 18, laparoscopic left adrenal giant pheochromocytoma resection was successfully performed under general anesthesia with CRRT support. After surgery, the patient exhibited favorable wounds healing and a significant reduction in plasma catecholamine metabolites to nearly normal level. Subsequently, the patient was discharged. All treatments described were administered with the patient's informed consent. During a 6‐month follow‐up, the patient remained asymptomatic with normal hormone levels.

### 2.2 CRRT treatment strategy

The patient underwent dialysis catheter insertion via the internal jugular vein. Continuous blood purification was conducted using Prismaflex (Gambro, Jinbao). The supporting hemodialysis filter and the hemofiltration replacement base fluid was provided by Chengdu Qingshan Likang Pharmaceutical Co., Ltd., National Medicine Zhunzi H20080452. The treatment was set to continuous venous–venous hemodiafiltration mode (CVVHDF) at a rate of 25–45 mL/kg/h, with a blood flow rate of 120–150 mL/min. Citric acid anticoagulation for CRRT was administered from June 4 to 9 at a dose of 180–220 mL/h, adjusted based on the serum calcium levels. On June 15, due to impaired liver function and a D‐dimer level exceeding 20 μg/mL, the anticoagulation method was switched to low molecular weight heparin sodium at a dose of 0.4 mL every 8 h. Surgical intervention was performed on June 18 without anticoagulation to reduce the risk of bleeding. The efficacy of CRRT treatments were recorded in Tables 1, 2, 3.

**TABLE 1:** Clearance of metanephrine analogs in plasma of patients treated with CRRT (from June 4 to June 12).

Monitoring items	Plasma metanephrine analog concentration (nmol/L)		
	3‐Methoxytyramine	Metanephrine	Methoxynorepinephrine
Treatment time			
Reference value	<0.18	≤0.50	≤0.90
3 days after CRRT treatment	0.31	2.08	>20.56
4 days after CRRT treatment	0.19	1.55	18.76
5 days after CRRT treatment	0.15	1.11	11.37
Stop CRRT for 6 h	0.21	1.62	18.06
Stop CRRT for 2 days	0.44	2.74	>20.56

*Note:* (1) Plasma metanephrine analogs are intermediate metabolites of catecholamines. (2) Plasma was not drawn to test metanephrine analog concentrations on Days 1–2 of treatment.

**TABLE 2:** Changes of hemodynamic indexes in patients before and after CRRT treatment (acute attack period from June 15 to June 18).

Treatment time	Blood pressure (mmHg)	Heart rate (times/min)	Breathe rate (times/min)	Central venous pressure (cmH_2_O)
Before CRRT treatment	210/113	120	32	20
4 h after CRRT treatment	138/91	126	20	15
8 h after CRRT treatment	101/77	117	18	12
16 h after CRRT treatment	95/66	112	12	9
24 h after CRRT treatment	101/68	112	17	8
48 h after CRRT treatment	90/60	114	17	5
72 h after CRRT treatment	112/79	102	12	7

**TABLE 3:** Changes of inflammatory factor levels, electrolytes, and heart, liver, and kidney function in patients before and after CRRT treatment (acute attack period from June 15 to June 18).

Treatment time	WBC (10^9^/L)	CRP (mg/mL)	PCT (ng/mL)	IL‐6 (pg/mL)	K^+^ (mmol/L)	Na^+^ (mmol/L)	pH value	Cr (umol/L)	ALT (U/L)	AST (U/L)	BNP (pg/L)
Before CRRT treatment	25.36	12.9	—	—	6.6	148	7.022	211	128	486	16,400
24 h after CRRT treatment	15.01	38.1	19.39	102.2	3.8	135	7.293	134	136	310	—
48 h after CRRT treatment	12.86	41.9	7.94	23.32	3.59	137	7.401	94	96	80	—
72 h after CRRT treatment	9.8	33.2	6.55	19.44	4.9	134	7.303	42	55	37	2483

Abbreviations: “—”, not detected; ALT, glutamic‐pyruvic transaminase; AST, glutamic‐oxaloacetic transaminase; BNP, brain natriuretic peptide; Cr, creatinine; CRP, C reactive protein; Il‐6, interleukin‐6; PCT, procalcitonin; WBC, white blood cell.

## 3 DISCUSSION

The typical clinical symptoms of pheochromocytoma usually include episodic headache, profuse sweating, and palpitations. However, the symptoms and signs can manifest atypically. Notably, pheochromocytoma crisis can lead to catecholamine cardiomyopathy, with a rapid onset and significant risk of fatality. This is particularly observed when the pheochromocytoma remains undiagnosed or poorly managed, resulting in severe outcomes such as multi‐organ dysfunction, acute left heart failure, and even cardiogenic shock. The initial hemodynamic instability complicates early management and escalates anesthesia‐related risks.^2^, ^3^ The cornerstone intervention for cardiogenic shock is ECMO, which has been utilized for life‐threatening undiagnosed pheochromocytoma‐related cardiogenic shock.^5^, ^6^ This approach significantly enhances survival rates in severe cases of pheochromocytoma‐associated cardiogenic shock. It is important to note that myocardial damage caused by pheochromocytoma is reversible, and timely intervention, including surgical treatment, offers curative possibilities.^7^ In this case, the patient presented with life‐threatening cardiogenic shock as the primary symptom, and was promptly rescued by ECMO. This not only prevented fatality but also facilitated subsequent treatments and increased the survival rate. However, the lack of sterility during emergency catheter insertion may lead to secondary bloodstream infection. Consequently, strict adherence to aseptic operation during invasive procedures, meticulous control of puncture site bleeding, regular dressing changes, and comprehensive observation for signs of infection are strongly recommended to prevent secondary infections that can complicate treatment.

In managing pheochromocytoma crisis with concurrent myocardial injury, the core therapeutic objectives include preventing shock and managing heart failure.^8^ Common strategies consist of using vasoactive agents, fluid resuscitation, and providing respiratory support.^9^, ^10^, ^11^ Crucially, assessing catecholamine levels and their metabolites in blood and urine serves as a highly sensitive and specific diagnostic indicator for pheochromocytoma.^12^ The significantly elevated concentration of methoxynorepinephrine also provides a hint for the diagnosis of pheochromocytoma. Furthermore, the test outcomes and clinical manifestations underscores the active secretion phase of the pheochromocytoma in patients. This phase easily exacerbates under external stimuli, triggering recurrent malignant arrhythmias that exert a huge influence on physiological function and engender swift deterioration. Conventional therapeutic approaches have certain disadvantages in attaining timely, effective, and consistent disease management. Numerous research studies have confirmed the effectiveness of continuous blood purification in swiftly, effectively, and extensively removing soluble inflammatory mediators, creatinine, and endotoxins from the systemic plasma through prolonged, gradual cardiopulmonary bypass. Additionally, continuous blood purification has been shown to improve inflammatory responses and multiple organ dysfunction.^13^, ^14^ Given that, CRRT is sequentially introduced following ECMO application. Table 1 shows a decrease in norepinephrine concentrations with extended continuous blood purification treatment duration. However, the suspension of CRRT leads to a marked rebound in hormone levels, significantly exceeding normal thresholds. Evidently, CRRT effectively eliminates catecholamine metabolites from patients. In addition to pheochromocytoma crisis, the patient also presented with sepsis, characterized by circulating toxins and inflammatory cytokines. As shown in Tables 2 and 3, the acute pheochromocytoma crisis induced cardiac, hepatic, and renal impairment, accompanied with circulatory collapse, pronounced hemodynamic oscillations, escalated inflammatory cytokine release, and intracellular acid–base imbalance. Subsequent CRRT intervention led to a progressive restoration in electrolyte ion levels (such as sodium and potassium), pH, cardiac, hepatic, and renal function markers, as well as inflammatory factors. This can be attributed to CRRT's role in removing toxins and suppressing inflammatory cascades, thereby mitigating the release and impact of harmful substances. By systematically clearing catecholamines and their derivatives, CRRT ultimately achieves hemodynamic equilibrium and promotes a balanced internal physiological environment. As a result, it extends the preoperative preparatory window and improves patient prognosis.

## 4 CONCLUSION

Continuous renal replacement therapy can be able to effectively eliminate catecholamines and their byproducts from the plasma, thereby promoting hemodynamic stability in patients. It notably enhances heart, liver, and kidney functions, reduces inflammatory cytokine levels, and extends the therapeutic window for patients.

## AUTHOR CONTRIBUTIONS

**Meinong Zhong:** Investigation; writing – original draft. **Yuanqiang Zhu:** Formal analysis; writing – review and editing. **Shaofang Wang:** Data curation. **Hengying Fang:** Methodology; software. **Guili Chen:** Conceptualization; funding acquisition; project administration; resources; supervision; validation.

## FUNDING INFORMATION

This study was supported by the Sun Yat‐Sen University Nursing Young Talents Cultivation Fund (N2020Y02).

## CONFLICT OF INTEREST STATEMENT

The authors declare that they have no conflict of interest to disclose.

## ETHICS STATEMENT

This case report study does not address human ethical issues. Patient and their families informed and consented to all treatments.

## CONSENT

Written informed consent for publication of their clinical details and/or clinical images was obtained from the patient. A copy of the written consent is available for review by the Editor‐in‐Chief of this journal.

## CARE CHECKLIST (2016) STATEMENT

The authors have read the CARE Checklist (2016), and the manuscript was prepared and revised according to the CARE Checklist (2016).

## Data Availability

The data and pictures designed in this case report can be found in the information system of the Third Affiliated Hospital of Sun Yat‐Sen University and the Jinyu Institutional Inspection Report.
